# *Daphnia magna's* Favorite Snack: Biofouled Plastics

**DOI:** 10.1002/etc.5393

**Published:** 2022-06-20

**Authors:** Lucy Polhill, Robyn de Bruijn, Linda Amaral‐Zettler, Antonia Praetorius, Annemarie van Wezel

**Affiliations:** ^1^ Institute for Biodiversity and Ecosystem Dynamics University of Amsterdam Amsterdam Netherlands; ^2^ Royal Netherlands Institute for Sea Research Den Burg Texel The Netherlands

**Keywords:** Microplastics, Freshwater toxicology, Bioavailability, Biofilm, Ingestion, Wastewater effluent

## Abstract

The influence of biofouling on zooplankton ingestion rates of plastics in freshwater environments has received limited attention. We investigated how biofouling of microplastics in wastewater effluent and in fresh surface water influences *Daphnia magna's* microplastic consumption. The differences in ingestion of the biofouled as compared with the virgin microplastics were higher for the surface water by a factor of seven compared with a factor of two for the effluent. The intake of biofouled microplastics by *D. magna* was higher compared with virgin plastics, but the reason for this preference should be further investigated. *Environ Toxicol Chem* 2022;41:1977–1981. © 2022 The Authors. *Environmental Toxicology and Chemistry* published by Wiley Periodicals LLC on behalf of SETAC.

## INTRODUCTION

An estimated 0.8–2.7 million metric tons of plastic end up in our rivers annually (Meijer et al., 2021). Plastics contaminate riverine and lacustrine food webs, and ingestion by freshwater species has been documented from filter feeders to apex predators (Santos et al., 2021). Toxicity, physiological effects, and physical deterioration of ingesting plastics has been reported for over 690 marine species, with more than 50 freshwater species showing clear allometric relationships (Jâms et al., 2020).

After macroplastic litter items enter the aquatic environment, UV radiation exposure and other physical forces can cause them to fragment into secondary microplastics (nominally between 1 and 1000 µm in size; Hartmann et al., 2019). Due to their small size, microplastics are bioavailable to a range of species across different trophic levels (Procter et al., 2019). Adverse effects of microplastic ingestion are reported at exposure concentrations higher than current environmental concentrations, such as reduced fertility, reduced feeding, weight loss, and increased mortality (Besseling et al., 2014). The factors driving ingestion of microplastics have been less extensively researched.

When microplastics enter the water, they develop a layer of biofilm, microbial assemblages containing eukaryotes, bacteria, and archaea (Zettler et al., 2013). This plastisphere is phenotypically diverse, but distinct microorganisms accumulating on plastics differ from surrounding planktonic communities (Zettler et al., 2013). Biofilm formation starts within minutes to hours after contact with water (Savoca et al., 2017), and might make the microplastics resemble a food source for zooplankton that is difficult to discern from their normal prey (Vroom et al., 2017). The biofilms contain prey items consumed by zooplankton and retain numerous different compounds including signaling molecules (Botterell et al., 2020). Bacteria and phytoplankton within the biofilm are capable of releasing chemicals that stimulate feeding activity of planktivorous species such as zooplankton (Bowley et al., 2021).

Zooplankton are crucial to healthy marine ecosystems as food sources for higher trophic levels and for nutrient and carbon cycling (Lin, 2016; Procter et al., 2019). Biofilm presence increases microplastic uptake by marine benthic filter feeders (Fabra et al., 2021). The influence of biofouling on ingestion rates of plastics by zooplankton has been studied largely in saltwater environments, whereas freshwater zooplankton have been less studied. Wastewater treatment plant (WWTP) effluent is a point source for microplastics to enter aquatic environments (Browne et al., 2011; Mintenig et al., 2017, 2020). Biofilm communities differ between microplastics incubated in surface waters versus wastewaters (Yang et al., 2020). Incubation in wastewater is reported to lower the toxicity of microplastics in comparison with freshwater (Schür et al., 2021). Thus, differences in ingestion between plastics from effluents or surface water might be an explanation for this. The aim of the present study was to investigate how biofouling of microplastics in wastewater effluent and in surface water influences ingestion by *Daphnia magna*. We tested the hypothesis that *D. magna* ingests biofouled microplastics at higher rates than virgin microplastics. Our aim was to increase our understanding of microplastic accumulation using lower microplastic concentrations and differing freshwater types than previous studies have employed.

## MATERIALS AND METHODS

### Materials

Water samples were taken at the Haaksbergen WWTP in the Netherlands in March 2021. We collected a 2‐L 24‐h averaged sample from the WWTP effluent located at 52°10′37.0″N 6°42′47.5″E, and a 2‐L surface water grab sample from the stream Bolscherbeek upstream from where the WWTP effluent is discharged at 52°10′37.1″N 6°42′45.5″E. Polyethylene microplastics were used as a representative and common polymer type in aquatic environments. A set of 63–75‐µm fluorescent polyethylene microbeads, with a density of 1.02–1.03 g/cm^3^ and a peak emission of 515 nm when excited at 414 nm, were purchased from Cospheric. Size and shape were confirmed by stereomicroscope and fluorescence using a Zeiss Axioskop 2 fluorescence microscope. The *D. magna* were cultured in tanks containing 2 L Aachener Daphnien Medium at 20 °C with a 16:8‐h light:dark cycle. Daphnids were fed yeast and 2.8 g of dried *Chlorella pyrenoidosa* three times/week, and the water was refreshed once a week. One‐month‐old female adults with an average size of 3 mm collected from the third brood cultured on the same date were used for the ingestion experiment. The *D. magna* were starved for 24 h prior to the ingestion experiment. The microplastic transfer was conducted in the fume hood to avoid contamination, and all materials used were disposed of in hazardous waste bottles. All laboratory equipment was cleaned with ethanol and Milli‐Q water prior to the experiments, and lids were placed on the tanks to prevent contamination.

### Biofouling

The effluent and the surface water samples were filtered over a 75‐μm stainless steel sieve. We placed 800 ml of WWTP effluent and 800 ml of surface water into separate 1‐ L beakers. For the experiment with biofouled plastics, we added 25 mg/L of the fluorescent polyethylene, corresponding to approximately 1.9 × 10^5^ microplastics/L. Any microplastics stuck to the surface were washed by use of a pipette to bring the plastics into suspension, and the beakers with microplastic suspension were sonicated. The beakers were placed on a rotator for 3 weeks under a 16:8‐h light:dark cycle at 20 °C, to generate a mature biofilm on the microplastic surfaces (Vroom et al., 2017). The water samples for the experiments with virgin plastics and for the control experiments were subjected to the same procedure (without the addition of microplastics during the 3‐week period). Microplastics were analyzed under both light and fluorescence microscopes to determine the presence of biofilm. Biofouled microplastics from both water types showed clear differences from virgin microplastics under the fluorescence microscope, that is, biofouled microplastics showed rough edges with organisms attached to the surface whereas virgin microplastics kept their original spherical shape. The filters used for the fluorescence microscope were a 450–490‐bandpass excitation filter and a 515–565‐bandpass emission filter. There was no autofluorescence due to chlorophyll detected.

### Ingestion

Directly after this 3‐week period, ingestion experiments for both the effluent and the surface water were performed with: (1) biofouled, (2) virgin, or, (3) no microplastics. For the biofouled microplastics treatment, the water samples containing 3‐week biofouled polyethylene microparticles were used without further dilution. For the virgin microplastics treatment, the polyethylene microplastics (25 mg/L) were added to the 3‐week‐old water just before the start of the experiment, and for the control treatments no microplastics were added. The ingestion was analyzed at five time points: 15, 30, 60, 120, and 240 min. Each exposure condition and time point were analyzed in triplicate, in 30 ml of exposure medium containing four *D. magna*/glass bottle, amounting to a total of 90 bottles and 360 daphnids. Each bottle was supplied oxygen during the exposure and covered with aluminum foil. During the whole ingestion experiment, no mortality was detected. The *D. magna* were studied individually. Daphnids were rinsed with Milli‐Q water and checked to see that no microplastics were stuck to the outside surface of the daphnids under the stereomicroscope. Thereafter, 1‐ml screw‐top Eppendorf vials were filled with deionized water and one *D. magna* each. The vials were homogenized by a Precellys 24 homogenizer at 5000 rpm for 10 s and then vacuum‐filtered with a Sartorius cellulose nitrate filter with a pore size of 0.45 µm. The microplastics on the filter were counted under a Leica M165C stereomicroscope with black light by placing a grid over the filter, to find the number of microplastics ingested/individual daphnid. The numbers of microplastics present in the daphnids were counted once they had been homogenized and filtered.

### Statistical analysis

All data were stored in Excel, and R was used to run statistical analyses. A two‐way analysis of variance (ANOVA) confirmed with a Tukey's post hoc test was run to compare ingestion rates for each water type and between the biofouled and virgin. The data from all time points were pooled to run the statistical analyses. The assumptions for the parametric ANOVA were met. The significance level for all statistical tests was defined as *α* = 0.05.

## RESULTS AND DISCUSSION

The *D. magna* easily ingested microplastics within the studied size range of 63–75 µm. The control group showed minimal contamination with microplastics present on the filter. No microplastics were present in the control daphnids as checked under the microscope. After filtration, however, some microplastics were seen on the filter also for the controls, which was corrected for.

**Figure 1:**
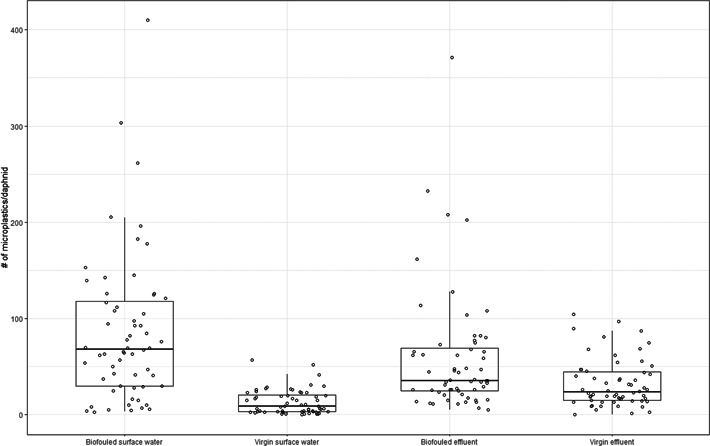
The average number of ingested microplastics for each treatment group, time points combined. Biofouled surface water–virgin surface water (*p* = 0.00); biofouled effluent–virgin effluent (*p* = 0.032); biofouled surface water–biofouled effluent (*p* = 0.037).

Biofouled microplastics were ingested more compared with the virgin microplastics (Figure 1), in line with previous studies using marine waters (Fabra et al., 2021; Vroom et al., 2017). The biofouling led to an increase of the mean ingestion rate for surface water by a factor seven, compared with a factor of two for effluent. Differences were statistically significant (ANOVA, *p* ≤ 0.05) for all exposure conditions. The preferential ingestion of the fouled plastics might be related to the presence of specific microorganisms in the biofilm or the specific infochemicals they produce (Lari et al., 2018; Rummel et al., 2017). Biofilm communities differ between surface water and wastewater (Parrish & Fahrenfeld, 2019). Dimethyl sulfide (DMS) is a chemosensory cue known to be able to influence ingestion by zooplankton species. Studies identified an increase in ingestion rates of DMS‐infused microplastics for saltwater zooplankton species (Botterell et al., 2020; Procter et al., 2019), but there is a lack of research on this aspect in freshwater systems. Microplastics aged in WWTP effluent were shown to induce a lower mortality in *D. magna* than microplastics aged in surface water (Schür et al., 2021), which is in line with the results of the present study.

**Figure 2:**
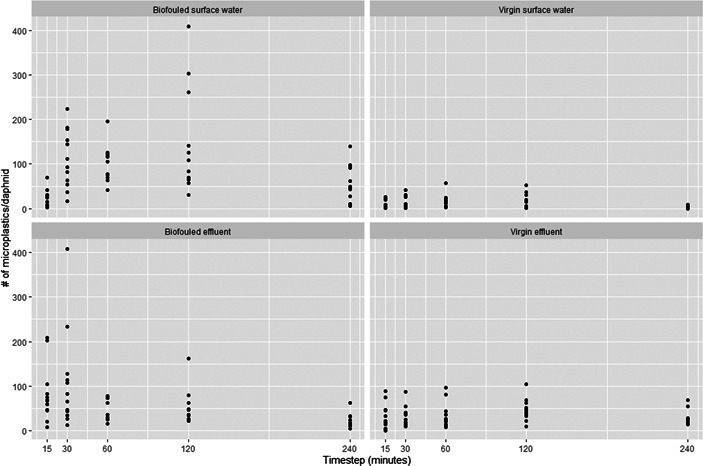
Number of microplastics ingested by individual daphnids/time step. The number of microplastics ingested/individual daphnid was measured per time step in ascending order: 15, 30, 60, 120, and 240 min. The highest individual rates in ingestion can be seen in the biofouled treatment groups. This figure shows the high discrepancies in individual ingestion rate/time point.

Figure 2 shows the number of microplastics ingested/individual daphnid/time step and exposure condition. Stereomicroscope analysis after the ingestion experiment showed that many of the daphnid guts were completely full of plastics. A plateau in the intake was reached after a short exposure duration (30 min), for both virgin and biofouled microplastics. The daphnids were starved for 24 h prior to the ingestion experiments. Therefore, they were likely to maximize their filter rate when initially exposed to the microplastics. Gut passage time ranged between 5 and 20 min. Therefore, it is likely that the daphnids ingested and egested microplastics between time points in the ingestion experiments. Larger individual variation in microplastic ingestion was, however, observed in exposure conditions with biofouling compared with the virgin microplastic.

Nominal instead of environmental concentrations of microplastics were used, due to the low volumes of each exposure medium used in this experiment. The concentration of 25 mg/L that we used is much higher than environmental concentrations (Mintenig et al., 2020), but is on the low end for earlier studies on ingestion of 63–75‐µm microplastics by zooplankton (Canniff & Hoang, 2018).

If ingested at higher concentrations (50–100 mg/L), microplastics were reported to negatively affect survival and reproduction of *D. magna* (Besseling et al., 2014; Ogonowski et al., 2016). Those studies show that microplastics are readily ingested by a variety of marine zooplankton species, which is consistent with the findings of the present study with the freshwater zooplankton species *D. magna*. Most marine zooplankton ingestion studies, however, do not include the presence of biofilm as a contributing factor to increased ingestion rates. The presence of biofilm and DMS are proven factors in increasing the number of microplastics ingested by zooplankton (Botterell et al., 2020; Fabra et al., 2021; Vroom et al., 2017).

The specific organisms within the biofilm itself were not identified but could be contributing to differing ingestion rates between surface water and effluent. When the biofouled microplastics were examined under the microscope, no differences could be discerned between the microplastics biofouled in the surface water and the effluent. The amount of biofilm present on the microplastics influences their density, which in turn can influence ingestion rates (Rummel et al., 2017). The polyethylene microplastics have a low density and tend to float on the surface; once biofouled, they have a higher tendency to sink. However, in our study the water column was mixed by the continuous aeration.

Zooplankton mistaking microplastics as their food source can impact the biogeochemistry and food availability in pelagic food webs (Galloway et al., 2017). Zooplankton species such as *D. magna* provide a fundamental link in the food chain, transferring energy and organic materials to higher trophic levels (Procter et al., 2019). Because microplastics ingested by *D. magna* have a strong likelihood of making their way up the food chain, the toxicity of biofouled microplastics also higher in the food chain should to be further investigated.

## CONCLUSIONS

Microplastics are prevalent in aquatic environments, making them easily ingested by a range of aquatic species. The presence of biofilm and water source were evaluated as factors that drive zooplankton microplastic ingestion. The *D. magna* were shown to readily ingest large numbers of 63–75‐µm polyethylene microplastics. The total number of biofouled microplastics ingested by *D. magna* for both water types was greater by a factor of three than the amount of virgin microplastics ingested. In addition, microplastics biofouled in effluent were ingested 45% less in comparison with the microplastics biofouled in surface water.

Our study shows that biofouled microplastics should be used in future microplastic ecotoxicology studies as more representative of plastics in the natural environment and demonstrating significantly different ingestion. Future studies should investigate further the factors driving microplastic ingestion by organisms in freshwater environments to support microplastic risk assessments and inform plastic waste management.

## Author Contributions Statement

**Linda Amaral‐Zettler**: Conceptualization; Supervision; Writing—review & editing. **Antonia Praetorius**: Conceptualization; Supervision; Writing—review & editing. **Annemarie van Wezel**: Conceptualization; Supervision; Writing—review & editing. **Lucy Polhill**: Conceptualization; Methodology; Formal analysis; Data curation; Writing—original draft; Visualization; Investigation. **Robyn de Bruijn**: Methodology; Formal analysis; Data curation; Supervision; Writing—review & editing.

###  

This article has earned an Open Data badge and an Open Materials badge for making publicly available the digitally‐shareable data necessary to reproduce the reported results. The data is available at https://doi.org/10.6084/m9.figshare.c.5648047.v1. Learn more about the Open Practices badges from the Center for Open Science: https://osf.io/tvyxz/wiki.

## Data Availability

Data follow the FAIR principles guidelines. The data repository can be found free of charge at https://doi.org/10.6084/m9.figshare.c.5648047.v1.
